# Using social networks to scale up and sustain community-based programmes to improve physical activity and diet in low-income and middle-income countries: a scoping review

**DOI:** 10.1186/s12966-023-01412-6

**Published:** 2023-01-27

**Authors:** Nina Abrahams, Sahar Khodabakhsh, Zoi Toumpakari, Frederick Marais, Estelle V. Lambert, Charlie Foster

**Affiliations:** 1 Centre for Exercise, Nutrition and Health Sciences, School for Policy Studies, Faculty of Social Sciences and Law, University of Bristol, Bristol, UK; 2 Health Through Physical Activity Lifestyle and Sport Research Centre, Division of Physiological Sciences, Department of Human Biology, Faculty of Health Sciences, University of Cape Town, Cape Town, South Africa; 3 Healthy Lifestyle Services, Public Health, Somerset County Council, Taunton, UK; 4 Africa Unit for Transdisciplinary Health Research, North-West University, Potchefstroom, South Africa

**Keywords:** Community, Programmes, Scale-up, Sustainability, Physical activity, Diet, Networks

## Abstract

**Background:**

Community-based programmes [CBPs], targeting increased physical activity and/or healthier eating, have been used in the prevention and management of non-communicable diseases. However, CBPs are only useful, insofar as they can be scaled up and sustained in some meaningful way. Social networks—defined as “social structures that exists between actors, individuals or organizations”—may serve as an important tool to identify underlying mechanisms that contribute to this process. This scoping review aimed to map and collate literature on the role of social network research in scaling-up and sustaining physical activity and/or diet CBPs in low-and middle-income countries [LMICs].

**Methods:**

Arksey and O’Malley’s framework and its enhancement were followed. Inclusion criteria were peer-reviewed articles exploring the role of social networks in scaled-up and/or sustained physical activity and/or diet CBPs in adult populations, published in English since 2000, and based in a LMIC. Databases searched were PubMed, Cochrane, Scopus, Web of Science, CINAHL, SocIndex, International Bibliography of the Social Sciences, and Google Scholar. Books, conference abstracts, and programmes focused on children were excluded. Two reviewers independently selected and extracted eligible studies. Included publications were thematically analysed using the Framework Approach.

**Results:**

Authors identified 12 articles for inclusion, covering 13 CBPs. Most were based in Latin America, with others in the Caribbean, the Pacific Islands, Iran, and India. All articles were published since 2009. Only three used social network analysis methods (with others using qualitative methods). Five main social network themes were identified: centralisation, cliques, leaders, quality over quantity, and shared goals. Contextual factors to be considered when scaling-up programmes in LMICs were also identified.

**Conclusions:**

This review has shown that the evidence of the use of social network research in programme scale-up has not yet caught up to its theoretical possibilities. Programmes aiming to scale should consider conducting social network research with identified network themes in mind to help improve the evidence-base of what network mechanisms, in what contexts, might best support the strengthening of networks in physical activity and diet programmes. Importantly, the voice of individuals and communities in these networks should not be forgotten.

**Supplementary Information:**

The online version contains supplementary material available at 10.1186/s12966-023-01412-6.

## Background

Community-based programmes [CBPs] may serve as an important approach in the prevention and management of non-communicable diseases [NCDs] [1, 2]. These refer broadly to programmes that engage a defined population in activities ranging widely from group-based interventions or mass media campaigns to environmental, structural or policy changes that are adapted to, set in, and ideally delivered by the community for that community [3]. However, CBPs are only useful insofar as they can be scaled up and sustained in some meaningful way. If the CBP does not reach enough people, then their effects are spread thin and the programme has less chance of making a sustainable and significant impact [1, 4–8]. There is, however, limited research on how to effectively scale-up and sustain complex community-based NCD prevention programmes [9, 10].

The authors chose to focus this review on low-income and middle-income countries [LMICs]. As community-based programmes are highly contextual it helps to focus on countries with a similar type of context to build more relevant data, particularly where this information may be lacking. The success of scaled-up programmes around the world is attributed, at least in part, to having government support and policies in place across multiple sectors (not just government or health organisations), as well as access to funding, and regular evaluation of how programmes operate in the real-world [10, 11]. However, the general dearth of scale-up literature is particularly pertinent in LMICs. For example, Reis et al. conducted a systematic review on scaling-up physical activity interventions [11]. The authors identified 16 scaled-up interventions in peer-reviewed literature, only two of which were based in an LMIC. While some evidence of scaled-up programmes exists in LMICs, they tend to significantly focus on HIV/AIDs, maternal health, and infectious diseases compared with NCD prevention [12, 13]. Of the two physical activity LMIC programmes identified by Reis et al., both were examples of practice-based evidence (evidence of effectiveness based on real world implementation as opposed to controlled trials) and heavily relied on the academic, government, and school networks to scale-up [11, 14, 15].

One potentially useful tool for understanding this role of networks and improving the theory and practice of the scale-up and sustainability of complex CBPs is social network research [SNR]. SNR is an example of practice-based research that examines the relationship between actors (individuals or organisations) in a system [16, 17]. The aim of SNR is to identify members of a particular network, their attributes, and their connections in the network and then to visually plot these relationships on a network graph [17]. These attributes and connections provide insight into the centrality of the network and the role or omission of key actors or organisations which may be critical to the success or vulnerability of the network. Hunter et al. conducted a systematic review and meta-analysis of 37 randomised controlled trials to identify the effectiveness of social network interventions over a range of health behaviours and outcomes [18]. The pooled evidence of sexual health studies (included in the meta-analysis) indicates that social network interventions result in improved health outcomes (OR: 1.51, 95%; CI: 1.27–1.81; *p* < 0.001) and are particularly useful for reaching and retaining underserved populations. However, of the 37 included interventions, only six studies were based in an LMIC.

This scoping review aimed to map and collate literature on the role of social networks in scaling-up and sustaining NCD prevention, physical activity, and diet community-based programmes in low-and middle-income countries. Our protocol paper reports the definitions of key terms and specific steps taken – available at https://bmjopen.bmj.com/content/bmjopen/11/9/e053586.full.pdf [3]. The findings of this review will help to determine the current scope of research and identify gaps in the literature.

The overarching research question of this review: *Is there research on social networks within scale-up studies of community-based physical activity and diet programmes in low- and middle-income countries? And if so, what is the nature of the role of social networks?*

## Methods

Our protocol paper reports the methods used for this review [3]. In summary, this review was planned around the methodological framework for scoping reviews outlined by the Arksey and O’Malley framework [19] as well as its enhancement [20]. Arksey and O’Malley propose five steps to a scoping review: 1) identifying the research question, 2) identifying the relevant studies, 3) study selection, 4) charting the data, 5) collating, summarising, and reporting the results, and an optional 6) consultation exercise. The sub-questions that guided the scoping review are included in Table 1. We followed the checklist provided by the Preferred Reporting Items for Systematic Reviews and Meta-analyses – extension for Scoping Reviews [PRISMA-ScR] [21]. The checklist includes 20 essential items which support methodological transparency (Additional file 1).

**Table 1:** Scoping review sub-questions

**Descriptive**	What is the volume of publications?
	What are the research designs of the publications?
	What is the geographical scope of the publications?
	Who are the publication authors?
**Social networks**	What types of networks and/or network interventions are described in the publications? What are they used for?
	Who is involved in the network(s)?
	What value, if any, do social networks bring to community-based programmes?
**Community-based programmes**	What types of community-based programmes are covered?
	What activities are included in the community-based programmes? Who is included? (age, sex, gender, health and economic status)
	Who is implementing these programmes? What settings are used for the programmes?
	What theories/theoretical approaches underpin the community-based programmes?
**Scale-up and sustainability**	What scale-up/sustainability theories are utilised in the publications?
	How is scale-up and/or sustainability conceptualised or operationalised?
**Mechanisms**	Are any potential mechanisms of scale-up and sustainability explored in the publications?

Inclusion criteria were: 1) being a diet and/or physical activity community-based programme; 2) being based in a low- and middle-income country 3) reports scale-up and/or sustainability outcomes; and 4) discusses social networks of the programme. Studies had to have been published in English since 2000. The cut-off was the date of the conducted search, 10 May 2021. No discrepancies to the protocol were made.

NTA carried out the searches of the electronic databases (PubMed, Cochrane, Scopus, Web of Science, CINAHL, SocIndex, and International Bibliography of the Social Sciences). The same search terms were used across databases; examples of terms include ("community based" OR complex) AND (intervention OR program*) AND ("social network*" OR "network analysis") AND ("scale up" OR sustain* OR disseminat*) AND (physical activity OR diet OR "non communicable") AND (LMIC OR Developing Countries OR Global South). A full list of search terms and strategy is provided in the protocol [3].

**Fig. 1:**
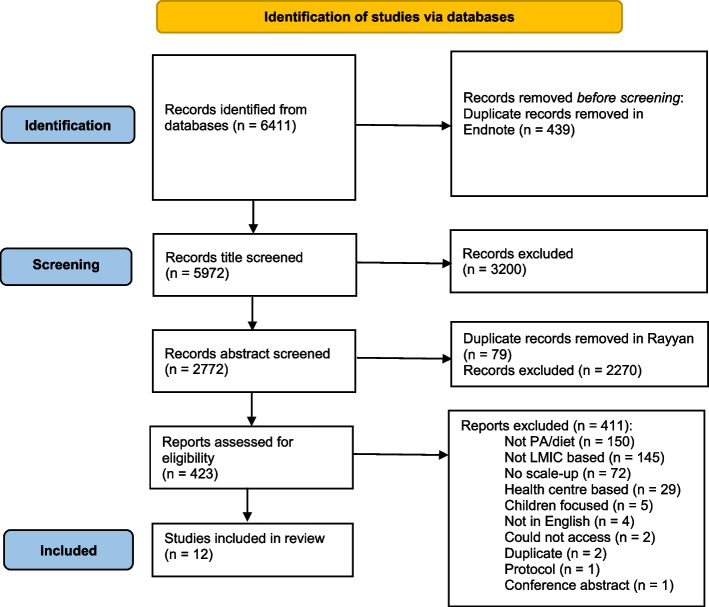
PRISMA flow diagram

In total, 6411 articles were identified. The titles and abstracts of these studies were extracted into the reference manager, EndNote X9, wherein 439 duplicates were removed. NTA conducted 100% of screening and reviewing of the remaining 5972 publications. In the first stage of screening (titles only), CF double reviewed the first 10% of titles to test for any reviewer discrepancies. The abstract and full text screening were then done using the Rayyan systematic review platform, a web-based tool for systematic review management [22]. For the second stage of screening (title and abstracts), 50% of the publications were double reviewed between NTA and one of three reviewers – SK, CF, or ZT. For the third stage of screening (full text), 15% of the publications were double reviewed by ZT. During, the title, abstract, and full-text double review process, no major discrepancies were noted, and so further double review was not deemed necessary. The review process is summarised in the PRISMA flow diagram below (Fig. 1).

After full text-review, included articles were thematically analysed for their network, CBP, and scale-up properties using the Framework Approach; a deductive qualitative analysis strategy that seeks to analyse data using pre-set categories [23]. This structured approach is useful in answering specific questions from a diverse body of literature, where one can identify themes from the outset whilst remaining flexible and true to the data. There are five stages in the framework approach [23]:

The first reviewer (NTA) first familiarised themselves with the data set by reading all articles, noting recurrent themes, and gaining an overall picture of the included publications. Then a thematic framework was identified which in this case was based on configurations of realist evaluation and the scoping review questions [3, 24, 25]. Thereafter two reviewers (NTA and SK) extracted the data using a customised data extraction form on Excel to guide the analysis (Table 2). This form was indexed into study details (e.g. author, year of publication), contextual factors, network factors, scale-up and/or sustainability outcomes, and any other underlying theories or mechanisms reported. NTA piloted the extraction sheet on the first five publications and refined it to improve applicability [20, 26]. NTA and SK then collated the data extraction information into a final excel chart to compare the programme, network, and scale-up/sustainability themes across studies. Network analysis factors, such as network size, density, degree centrality, and network stakeholder attributes were not reported across studies – limiting analysis along network concepts [27]. The findings outlined in the data extraction chart were therefore thematically analysed based around clustering of common concepts found in social network analysis research and scale-up literature [17, 28, 29].

**Table 2:** Data extraction form – headings in Excel

**Article details**	Study ID First 3 and last author No. of authors Year Title Country Continent LMIC Area (if given) Journal/publisher Type of article (journal (original article), editorial etc.) Type of article e.g. primary (qual / quant), framework, theory
Context	Description of CBP Who does CBP serve? CBP setting Name of CBP (if given) Any other contextual factors Intervention time
Social network descriptors	Description of network structure Media vs person List of actors Comments on networks Is SNR mentioned explicitly?
Physical activity / diet	PA (yes/no) Diet (yes/no) Other?
Outcome	Description of CBP outcomes Description of scale-up outcomes Vertical or horizontal Description of sustainability outcomes Scaled-up or proposed plans? Theories used
Mechanisms	Possible underlying mechanisms
Other comments	

## Results

The scoping review process identified 12 publications for inclusion – namely, articles that explored and discussed the importance of social relationships between network stakeholders in a scaled-up community health programme. Articles covered 13 unique community-based programmes (three articles spoke to more than one programme) spanning 7 countries or regions. Most programmes [8] were based in Latin America (such as Colombia, Brazil, Mexico), with other programmes taking place in the Caribbean, the Pacific Islands (Tonga, Federation of Micronesia), and Asia (Iran, India). All articles were published during or after 2010, except one published in 2009. Programmes focused on physical activity (*N* = 8), diet (*N* = 2), and both diet and physical activity (*N* = 3). A summary of included studies is provided in Table 3, a short description of each programme and their scale-up or sustainability achievements is provided in Additional file 2.

**Table 3:** Summary of included studies

CBP #	Programme name	First author and year	Country	Population and setting	Target outcome	Scale-up and/or sustainability achievements	Publication research methods used
1	Alianza por la salud alimentaria (Alliance for nutritional health)	Huang et al. 2015 [30]	Mexico	General population; mass media and academia	Diet	Influenced the tax policy in the country	Case study
2	Redcolaf	Parra et al., 2011 [31]	Colombia	Government, organisations, and academia; PA network of Colombia	PA	Influenced government policy to focus on physical activity	Social network analysis
3	Ciclovia Recreativa (Open Streets)	Meisel et al., 2014 [32]		General population; public spaces	PA	Maintained over time, regular events, and is the largest Open Streets programme in the world	Social network analysis
4	Healthy Habits and Lifestyles Program (HEVS)	Díaz del Castillo et al., 2017 [33]; Rubio et al., 2021 (merged) [34]		General population; public spaces	PA	Maintained over time, scaled-up and funded on a national level, operating in most of the country	Interviews, survey
5	Recreovia			General population; public spaces	PA	Widely used programme operating in 95% of Bogata’s hubs. Institutionalised in government	
6	Academia da Cidade program (ACP)	Paez et al., 2015 [35]	Brazil	General population; public spaces	PA	Recognised as a government initiative. Paved the way for a nationally scaled programme	Interviews
7	CuritibAtiva	Ribeiro et al. 2010 [36]		General population; community-based	PA	Maintained over time	Discussion groups
8	Guide for Useful Interventions for Activity (GUIA) Project	Parra et al., 2011 Pratt et al., 2010 [37]; Brownson et al., 2010 [38]	Latin America	Government, organisations, and academia; PA network of Brazil and Latin America	PA	Created sustained networks. Paved the way for a nationally scaled programme	Social network analysis
9	Healthy Municipalities, Cities, and Communities Strategy (HMCS)	Chaparro et al., 2020 [39]	Caribbean	General population; local authorities and related sectors	PA & diet	Used in multiple countries	Qualitative systematic review
10	Pacific Diabetes Today Coalitions	Aitaoto et al., 2009 [40]	Pacific Islands	General population; community-based	PA & diet	Continued after official funding ended	Interviews
11	Pacific Sports Partnerships’ (PSP) sports-for-development program	Keane et al., 2019 [41]		General population, disability; sports sector	PA	Contributed to national health strategies	Interviews, focus groups, observations
12	Isfahan Healthy Hearts programme	Sarrafzadegan et al., 2018 [42]	Iran	General population and high-risk groups; mass media and public spaces	PA & diet	Inspired similar projects and contributed to national health strategies	Interviews
13	Nutrition communication	Gavaravarapu et al., 2014 [43]	India	General population and women with children; mass media and community-based	Diet	Maintained over time. Is run through government mandates	Interviews

Three studies used social network analysis methods [31, 32, 37]. One publication used mixed methods: interviews and a survey [33]. Eight publications used qualitative methodology. All the publications collected data at a single time point. Limited reference was made to social network theory in the articles. In addition, while evaluation was deemed important for programme implementation and scale-up, no specific reference was made to scale-up definitions, theories, or frameworks. Only a brief reference to horizontal scaling was indicated in three publications – that of social marketing [42], opinion leader theory [43], and strategies to increase public demand [30]. Only one sustainability theory was referenced in two of the included articles [33, 40]; that of Shediac-Rizkallah and Bone; and Scheire.

### Social network descriptors

Five broad social network themes were thematically generated from the 12 studies: centralisation, cliques, leaders, quality over quantity, and shared goals.

#### Centralisation of the network

Two network structures were described in the publications. The first was a centralised network, typified by a few highly connected organisations at the ‘centre’ of the network with less tightly connected partners around these central actors [29]. This type of network structure was seen in the social network analysis of physical activity-focused organisations in Colombia [31].

Alternatively, other publications described the network of their programme as being decentralised; characterised by a flatter network structure wherein most organisations have a similar number of ties instead of a few central players [29]. This structure was seen in the physical activity-focused organisations in Brazil [36, 37]. Riberio et al. hypothesised that the decentralised network of CuritibAtiva (operating from multiple local administrative hubs) created opportunity for collaboration with both the formal and informal sector and public sector; however, the lack of central leadership made it more difficult to mobilise across institutions [36].

#### Cliques

The social network publications found that organisations in the network often clustered together (had closer relationships) with organisations of the same sector type or geographical location. For example, Ciclovia Recreativa health organisations clustered together and non-health sector organisations clustered together [32]. In Brazil, organisations that were part of Project GUIA were more likely to collaborate than organisations that were not Project GUIA members [38]. The GUIA project (Guide for Useful Interventions for Activity in Brazil and Latin America) was facilitated by the Centers for Disease Control and Prevention World Health Organisation Collaborating Center for Physical Activity and Health. One of the primary objectives of the project was to “establish and build cross-national, collaborative relationships with researchers, practitioners, and institutions in Brazil to enhance capacity to determine and implement evidence-based interventions that promote physical activity”, it is therefore not surprising that there was greater likelihood of collaboration [37]. Although the opposite was found in Colombia where organisations in Redcolaf were less likely to collaborate than organisations outside of the Redcolaf network [31]. In both Brazil and Colombia, organisations were clustered around others that were geographically proximal and around areas that have access to resources – such as the south region of Brazil where more nationally and internationally recognised research institutions are based [31, 38]. The social network analyses also found that research organisations were more likely to collaborate than practice-based organisations. This was thought to be because practice-based organisations are more likely to compete for funding and so be focused on their own outcomes [31].

The qualitative publications acknowledged that such clustering is not always ideal, and that having health experts and CBP champions diffused across different health and non-health sectors is preferable [14, 30, 33, 35, 39]. The authors argue that this diffusion increases the ability to take advantage of multiple windows of opportunity to implement and spread the programme.

#### Leaders

Perceived leaders in the programme networks were shown to have more connections and provide important scale-up and sustainability functions. The social network analyses in Colombia showed that the more ‘important’ an organisation was deemed (indicated on the social network survey as “relevance of the organisations participating in Ciclovía”), the more integrated they tended to be [31, 32].

Government representatives, organisations providing funding or that were able to source financial support, the academic sector, and community groups were highlighted by the publications as leaders in the programme networks. For example, in Colombia, continuity of the network was attributed to the high profile of the government agencies involved in the network, where nearly 80% of organisations work in the government sector [31]. Similarly, the HEVS programme (Hábitos y Estilos de Vida Saludable; Healthy Life Habits) has a secure network with dedicated government policy support and committed funding from multiple avenues which protects them from changes due to administrative cycles [33]. Multiple programmes highlighted the importance of continued government support in sustaining the programme [34, 35, 39, 42, 43], as well as the need for sustained funding [33, 35, 40, 41]. The HEVS programme also mentioned the importance of in-kind collaboration (organisations that provide resources) to sustain the network [33] as well as leaders who are able to source multiple funding streams [41].

Multiple publications also mentioned the importance of collaboration with academic and research institutions who can provide technical support, evaluation of programmes, and dissemination of information; as well as collaboration with organisations that have the skills to implement the programmes [30, 33–37, 39, 42]. Using pre-existing structures, organisations, and networks was particularly useful for implementing and scaling up the programmes [37, 39, 40, 42]. Two publications highlighted mobilised communities as important stakeholders in the network to grow and sustain community-based programmes [30, 34].

The LMIC setting of the programmes seemingly affected who could be a leader. Often these programmes were reliant on funding, resources, and skills training from high-income countries [14, 40, 41]. For example, of the six stakeholders in the Pacific Sports Partnerships programme, three of the stakeholders (the programme funders, managers, and designers) were based in Australia. In contrast, the implementers, ad-hoc contributors, and participants were based in Tonga. LMIC programmes might also be less able to rely on volunteer leaders in the network. Authors noted that in a context of a low-income setting it was difficult to maintain enthusiastic people to lead and implement the programme without providing payment and job security [31, 33, 41].

#### Quality over quantity

The strength and quality of the relationships in the network were deemed more important than the number of people in the network. Both HEVS and Recreovía in South America, and in examples of Nutrition Communication in India, the programmes limited the number of champion trainers that implement the programme so that they could afford to provide continuous training and payment for the trainers [33, 43]. In the Pacific Islands, Indian, and Recreovía programmes, the authors recognised the importance of trusted, local implementers who were culturally sensitive when advertising and conducting programmes [40, 41, 43]. Authors highlighted the importance of committed and enthusiastic champions as integral to the network to motivate communities as well as be the trusted voice for community needs [33, 34, 40].

#### Shared goals

Authors reported that having shared goals and vision between organisations in the network helped in scaling-up and sustaining programmes [36, 37, 40, 41]. For example, the CuritibAtiva and Pacific Sports Partnerships programmes found that different organisations in the network had different agendas that were not clearly communicated which led to working in isolation and so wasted time and resources [36, 41]. Comparatively, the Pacific Island programmes that were sustained occurred when organisations shared the same overall goals and missions [40, 41].

Shared goals were also found to moderate network integration, but not always in the same manner. For example, the network analysis in Colombia found that the longer an organisation was with Ciclovia, the less integrated they were in the network [32]. The authors propose that this may be because older organisations prefer to maintain status quo and so are less likely to collaborate with new partners. In comparison, newer organisations form ties to learn and develop in the network [32]. Alternatively, organisations situated in the Brazil network for longer were more likely to be connected to others [38]. The authors hypothesise that this was due to the moderating effect of stakeholders having the specified shared goal of increasing networking – regardless of time in the network.

Shared goals are a particularly pertinent theme in LMICs. Multiple authors spoke to the high NCD burden in LMICs [33, 34, 40, 43]. However, they also found that health systems and policy in these settings were often not yet focused on NCD prevention – making it difficult to mobilise relevant funders and stakeholders [37, 39, 42]. Authors also considered the strong traditional cultures that are often prominent in LMICs. For example, the high rates of subsistence farming or religion need cultural considerations in developing and implementing programmes [41, 43]. Huang et al. highlighted the importance of developing messages and shared goals around what is important to the general population [30]. For example, the messaging of the Alliance for Nutritional Health was tailored around diabetes prevention as that was topical in the Mexican population. Tonga used sports federations as it is known as being a sport-loving nation [41], while other programmes in South America have focused their messaging on physical activity dance classes that are more culturally relevant – although predominantly to women [33–35]. Social media was described as a useful tool to create and maintain shared values by widely disseminating information and programme support across and beyond the network [30, 33, 35, 42].

## Discussion

This scoping review aimed to map and characterise published literature on the role of social networks for scaling-up and sustaining NCD prevention, physical activity, and diet CBPs in LMICs. We identified 12 publications for final inclusion, covering 13 unique community-based programmes. Only three publications conducted a social network analysis. This meant that network analysis factors, such as network size, density, degree centrality, and network stakeholder attributes were not reported across studies – limiting analysis along network concepts [27]. Most of the included articles used qualitative methods, such as interviews and focus groups, to describe the experiences of network stakeholders in implementing community programmes at scale with reference to network relationships. It was therefore deemed appropriate to do thematic analysis for this scoping review. Five broad social network and scale-up themes were generated: centralisation, cliques, leaders, quality over quantity, and shared goals – that could be considered when scaling up CBPs.

One of the included programmes, Project GUIA, representing a physical activity inter-sectoral network in Latin America, particularly highlighted the importance of having strong networks. This network included high government and academic support for active research and dissemination of physical activity programmes [14, 37]. The success of this network at its peak can be starkly seen in this review where half of the included publications are based in this region. However, while acknowledging the importance of multi-sector collaboration, the World Health Organisation states that without understanding network properties, collaboration can be labour intensive and time consuming and can lead to confusion, duplication of efforts, or inaction [29, 44].

For example, understanding the centralisation of a programme network might help determine its strengths and weaknesses. A more centralised network can be easier to coordinate and allow for greater alignment of activities, direction, and accountability. A decentralised network may be fragmented, politically weak, or waste resources by duplicating activities [44–46]. However, a decentralised network has greater potential for local outreach and flexibility to local needs and intersectoral collaborations compared to a central system where central organisations might be less likely to know what other related outside the network organisations are doing [29, 45, 46].

The strength and capability of partnerships might also be affected by who is connected in a network. Organisations in a network tend to collaborate with other organisations of similar network type and geographical proximity – forming clusters or cliques. The use of social network analysis to identify cliques and their effects has been used especially in research on adolescent peer influence [47, 48]. In these groups, it’s found that adolescents who are similar and in geographical proximity are more likely to create friendships together than with people who are dissimilar or further away. Adolescents in the same clique are also more likely to trust each other, share ideas, and influence each other’s behaviour. This scoping review found a similar network structure among organisations wherein organisations across sectors (e.g. academic and practice, different government departments, private vs public, health and non-health, different geographic locations etc.) were less likely to be connected [31, 38]. Both the literature and the publications from this review indicated that it is ideal for programme representatives or champions to be diffused across different sectors and spaces to increase diversity in thinking, scope for programme advocacy, mobilisation of resources, and more chance of taking advantage of windows of opportunity [30, 33, 49–51] – particularly with the help of ‘bridging’ persons and leaders who create connections between these cliques [12, 14, 29, 30, 33, 35, 39, 52].

Having shared goals was highlighted as one way to connect within and between different sectors. For example, networks with a shared and emphasised goal of *networking* are more likely to have stronger, more diverse, and higher quality collaborations than in those with different priorities [31, 36, 38]. This can be seen in the academic setting where collaboration is often perceived to be rewarded [53] – compared to practice-based organisations who are more likely to be competing for funding [31, 49].

However, the results of the review also highlighted the tension between sharing goals and tailoring evidence to individual stakeholders. Personalised story-telling and relevant evaluation outcomes are important for scale-up and sustainability [30, 41, 54]. Network leaders need to send tailored messages out to the right people to increase buy-in and commitment of more partners. In an Australian review of physical activity programmes, Koorts et al. identified that while some stakeholders are convinced by evidence of programme effectiveness on a target outcome, others prefer evidence from experiences in other settings, by perceived credibility or visual evidence of programme enjoyment, or by personal support of the underlying programme theory [54].

As found in the included publications and corroborated by the World Health Organisation ExpandNet scale-up framework, quality and strength of relationships in a community programme should not be sacrificed when trying to achieve greater reach and more network connections [29]. Network research in the workplace has shown that increases in network connections (quantity) can create more opportunities for finding support to achieve goals [55]. However, if the network grows too large, this can overwhelm the capacity of the system and cognitive load of individuals – reducing collaboration, helping behaviour, and network performance. This interaction is moderated by quality of relationships, whereby the more people know about each other, trust each other, and understand how they are able to help, the more they can continue supporting each other even in large networks [55].

This scoping review identified additional contextual considerations for LMICs. For example, the lack of findings in low-income countries may be because LMICs tend to still have high rates of infectious disease and maternal and child ill-health, and a political focus on delivering basic healthcare. As a result, government priorities and policy may not yet support the scaling of NCD prevention in community-based programmes [51]. The lack of policy support and infrastructure in lower-income countries means that NCD prevention programmes are often reliant on international funding and expertise [51, 56, 57]. This makes these programmes particularly vulnerable to not only internal but also external administrative and funding changes [58]. Another consideration of programmes in a low-resource setting such as LMICs is that stakeholders, particularly programme implementers, might also expect to be renumerated (financially or otherwise) for their time as they may not be able to afford to volunteer [59]. This may create tension between cost, priorities, and stakeholder availability needed to implement and expand a programme.

While the current review focused on LMICs, considering the general dearth of research on scale-up of health programmes using social networks [27], the findings of this review may be useful to settings more globally. Contextually, low- and high-income countries can differ in many ways. For example, there is a longer history of community-based health programme implementation in high-income countries with less overlap between infectious and non-communicable disease burdens compared to low-income countries [60] – which may influence buy-in and capacity of local stakeholders to implement community programmes. However, the social network themes discussed in this review, such as thoughts around the benefits and limitations of centralisation, having shared goals, and what makes a leader can be similarly found in research in high-income settings [29, 54, 61]. This highlights that network lessons can be learnt across borders; however, it is integral to understand how context affects network development, scale-up, and sustainability.

### What network information is missing?

This review identifies a range of social network themes in the scale-up of community-based programmes. However, there are other network considerations in the literature that were not captured in this review but may be important, nonetheless. For example, none of the publications are based solely on an online social network platform. This could indicate that face-to-face activities are essential for scaling-up programmes. Alternatively, it may represent untapped potential in low- and middle-income countries where access to the Internet and mobile devices is growing [27, 62]. The role of individuals was also less explored. Namely, the influence of personalities and types of people *within* organisations that influence between-organisation networks [63]. Another limitation of the included social network analyses is that they did not consider the voice of the communities that take part in the programmes. Understanding the ‘top-down’ structures of governments, programme funders, and designers is important as they offer accountability, funding, and coherence to the CBPs [30, 39, 41]. However, ‘bottom-up’ decentralised communities that advocate for what they want ensures that programmes are appropriate and sustained [30, 33, 34, 64]. Therefore, including their perspective in the overall social network of the programme is integral. The lack of individual and community voices found in this review reflects the general lack of research across multiple levels in network research [65, 66]. Additionally, the evidence supported the understanding of the role of social networks in sustaining or scaling community-based programmes; however, there was little evidence as to the type of social network *interventions* that can be implemented to improve the programmes [18, 67].

### Strengths and limitations

This is the first review to examine the complexity of scaling-up community-based programmes in LMICs with the potential for social network research. While a limited number of articles was identified, the review highlights network and contextual properties that can be useful in developing this field of research further, particularly in middle-income countries where the publications were based.

There are various limitations in this review. Scoping reviews do not aim to critique the included publications’ methodology and so cannot make claims about the validity of the studies methods and findings [20]. Publications in a language other than English, as well as community-programmes focused on children, were not included as this was beyond the timeline and scope of this review. In addition, the wide range of potential terms used in the literature, such as ‘scale-up’ vs ‘dissemination’ vs ‘implementation’, [10] ‘scalability’ and ‘spread’ [68] and the limitation on databases searched may mean that publications were missed. To mitigate these limitations, there was continuous engagement with the literature and among authors to refine the terms and multiple reviewers were used to analyse the publications to increase reliability and credibility of the research [20, 26]. This review focused on mechanisms in relation to social networks; however, there are a variety of other possible mechanisms at play in the complex process of scaling-up and sustaining community-based programmes [54].

## Conclusions and future research

Social network research could serve as an important tool for understanding and improving the scaling-up and sustaining of community-based physical activity and diet programmes in LMICs and settings more globally [27, 63, 66]. However, this review has shown that the evidence has not yet caught up to this theory. Five network themes were identified that may be useful in understanding how CBPs operate and can be scaled. Community programmes aiming to scale should consider conducting social network research with these themes in mind to help improve the evidence base of what mechanisms, in what contexts, might best support the strengthening of networks in physical activity and diet programmes. Importantly, the voice of individuals and communities in these networks should not be forgotten.

## Supplementary Information

**Additional file 1.**

**Additional file 2.**

## Data Availability

The datasets used and/or analysed during the current study are available from the corresponding author on reasonable request.

## Abbreviations

CBP: Community-based programme
NCDs: Non-communicable diseases
LMIC: Low-and middle-income country
SNR: Social network research
OR: Odds ratio
CI: Confidence interval
PRISMA-ScR: Preferred Reporting Items for Systematic Reviews and Meta-analyses – extension for Scoping Reviews
NTA: Nina Abrahams
CF: Charlie Foster
SK: Sahar Khodabakhsh
ZT: Zoi Toumpakari
GUIA: Guide for Useful Interventions for Activity in Brazil and Latin America
HEVS: Hábitos y Estilos de Vida Saludable; Healthy Life Habits
FM: Frederick Marais
EVL: Estelle V. Lambert
