# Supplementary material for: Using social networks to scale up and sustain community-based programmes to improve physical activity and diet in low-income and middle-income countries: a scoping review
Source: Int J Behav Nutr Phys Act. 2023 Jan 27;20:8. doi: 10.1186/s12966-023-01412-6 (PMC9883854; doi:10.1186/s12966-023-01412-6)
Supplement: Supplementary file 2 — Additional file 2. [file 12966_2023_1412_MOESM2_ESM.pdf]

| Additional File 2. Short description of each programme and their scale-up or sustainability achievements | |
|---|---|
| **Programme name and country** | **Programme descriptions** |
| **Physical activity programmes (8)** | |
| Redcolaf – Colombia | Redcolaf is the Colombian Physical Activity Network that aims to increase physical activity at a population level. The consortium has pushed for relevant government policy changes to increase focus on physical activity and legitimise physical activity programmes in Latin America (Díaz del Castillo et al., 2017). |
| Ciclovia Recreativa (Open Streets) – Colombia | Ciclovía is a multisectoral community-based programme in which streets are temporarily closed to cars and similar transport, allowing exclusive access to individuals for leisure activities and physical activity. Open Streets runs all over the world; however, the Ciclovía in the capital of Colombia, Bogota, has a long history (since 1974), runs weekly, and is the largest Open Streets programme in the world. |
| Healthy Habits and Lifestyles Program (HEVS) – Colombia | Community-based program offering free physical activity classes by trained instructors every week (e.g., rumba, folklore, aerobics, flexibility, martial arts) in public spaces (parks, plazas, streets, malls, and community centres). This programme had been running for over 13 years and has been since scaled-up and funded on a national level and operates in 19 of the 32 Colombian departments. It has also increased its mandate to include healthy eating and smoke-free environments. |
| Recreovia – Colombia | Similar to HEVS. The programme started in 1995 as a complementary activity to Ciclovía. It went from running in one hub with low attendance to becoming a widely use, independent programme operating in 95% of Bogata's hubs. It is maintained and institutionalised by government entities. |
| Academia da Cidade program (ACP) – Brazil | Similar to Recreovia and HEVS. The ACP programme was institutionalized in 2002 in Recife, Brazil and is recognised as a health secretary initiative. It paved the way for the national programme, Academia da Saude, which has been implemented in 4,000 cities across the country since 2010. |
| CuritibAtiva – Brazil | Based in Curitiba, Brazil, this programme aims to inform and educate city residents about the potential for physical activity to improve quality of life. Outputs includes the distribution of printed educational materials addressing physical activity, assessment of lifestyle practices, and physical fitness examination. It runs parallel to other health programmes in the area since 1998. |
| Guide for Useful Interventions for Activity (The GUIA Project) – Brazil and Latin America | Multi-partner research partnership between local and international (United States) academic and governmental institutions – funded by the Centers for Disease Control and Prevention [CDC]. The GUIA Project aimed to create a network and infrastructure for surveillance, evaluation, and public health programming for non-communicable diseases. The project resulted in establishing a strong network between research and public health practice and effective dissemination and replication of relevant health programmes in Brazil and Latin America. Research from the project directly resulted in one of the largest national scale-ups of a physical activity promotion programme in the world, Academia da Saúde (Parra et al., 2013). |
| Pacific Sports Partnerships' (PSP) sports-for-development program – Pacific Islands | Since 2009, the Australian Government has provided funding and development resources to seven national sporting federations under the PSP program in Tonga (such as netball, hockey, swimming, and rugby). The most successful programmes have received local funding and have contributed to the national strategy on non-communicable |

| | |
|---|---|
| | diseases. However, the programme is still largely sustained through international funding. The PSP programme has since rebranded to become TeamUp. |
| **Diet programmes (2)** | |
| Alianza por la salud alimentaria (Alliance for nutritional health) - Mexico | This is a consortium of academics and more than 20 civil society organisations in Mexico. The group engages in various activities around nutritional health such as academic conferences and mass media public awareness campaigns. The pressure of their campaigns resulted in the introduction of a tax on sugary drinks and junk food in the country. |
| Nutrition communication – India | This publication explores three case studies of nutrition communication in India. All three of these nutrition communication strategies (e.g., nutrition education, mass media campaigns) have been sustained since the 1980s. Two are run through government mandates. |
| **Diet and physical activity programmes (3)** | |
| Healthy Municipalities, Cities, and Communities Strategy (HMCS) – Latin America and Caribbean | Developed by the Pan American Health Organization in 1990. Most HMCS programs are focused on physical activity and nutrition lifestyle programmes and services, such as vaccination campaigns or screening for chronic diseases, as well as tobacco cessation. Eighteen countries across Latin American and the Caribbean currently run health projects under the HMCS banner. |
| Isfahan Healthy Hearts programme – Iran | This programme ran from 2000 to 2007. It consisted of 10 multidisciplinary interventions that aimed to promote healthy lifestyle behaviours including healthy nutrition, tobacco control, physical activity, and stress management, with the aim of ultimately preventing cardiovascular disease and non-communicable diseases more generally. Key strategies for intervention activities included public education through mass media and intersectoral collaboration. The programme has inspired similar projects in other parts of Iran and has been used as a reference for policy planning. |
| Pacific Diabetes Today Coalitions – Pacific Islands | In 1998, the Pacific Diabetes Today Resource Center, in collaboration with the CDC, provided training, technical assistance, and funding to diabetes coalitions in 11 Pacific Islands communities. Nine of the 11 coalitions continued to provide programmes four years after funding ended in 2004. This includes two programmes in the lower-middle income islands of the Federation States of Micronesia. The Chuuk Women's Council and the Kosrae Diabetes Today Coalition. |
